# Recent Advances in Wearable Thermal Devices for Virtual and Augmented Reality

**DOI:** 10.3390/mi16040383

**Published:** 2025-03-27

**Authors:** Minsu Park

**Affiliations:** Department of Polymer Science and Engineering, Dankook University, 152 Jukjeon-ro, Yongin 16890, Gyeonggi-do, Republic of Korea; minsupark@dankook.ac.kr

**Keywords:** virtual reality, augmented reality, wearable devices, thermal feedback, thermal actuators, cutaneous thermoreceptors

## Abstract

Thermal technologies that effectively deliver thermal stimulation through skin-integrated systems and enable temperature perception via the activation of cutaneous thermoreceptors are key to enhancing immersive experiences in virtual and augmented reality (VR/AR) through multisensory engagement. However, recent advancements and commercial adoption have predominantly focused on haptic rather than thermal technology. This review provides an overview of recent advancements in wearable thermal devices (WTDs) designed to reconstruct artificial thermal sensations for VR/AR applications. It examines key thermal stimulation parameters, including stimulation area, magnitude, and duration, with a focus on thermal perception mechanisms and thermoreceptor distribution in the skin. Input power requirements for surpassing thermal perception thresholds are discussed based on analytical modeling. Material choices for WTDs, including metal nanowires, carbon nanotubes, liquid metals, thermoelectric devices, and passive cooling elements, are introduced. The functionalities, device designs, operation modes, fabrication processes, and electrical and mechanical properties of various WTDs are analyzed. Representative applications illustrate how flexible, thin WTDs enable immersive VR/AR experiences through spatiotemporal, programmable stimulation. A concluding section summarizes key challenges and future opportunities in advancing skin–integrated VR/AR systems.

## 1. Introduction

Virtual reality (VR) and augmented reality (AR) are advanced technologies that enhance human interaction through high-fidelity user interfaces, enabling real–time multisensory engagement, including visual, auditory, haptic, and olfactory stimuli [1,2]. One key to qualitatively enhancing immersion in a VR/AR environment is thermal stimulation. While research advancements and commercial adoption of haptic interfaces have preceded those of thermal interfaces due to their greater technological maturity and more direct impact on user interactions, thermal feedback has demonstrated its effectiveness as an alternative to vibrotactile mechanisms in environments where vibratory noise introduces confounding effects [3]. The rapid advancement of thermal technologies is expected to expand VR/AR applications across various industries, including entertainment [4,5,6], education [7,8], healthcare [9,10], and training [11,12,13].

An important focus is on skin-integrated systems as a foundation for generating mechanical and thermal stimulation by effectively activating afferent nerves or engaging cutaneous receptors. The thin, flexible, and stretchable design of these systems enables seamless integration onto various body regions [14,15], and their ability to deliver spatiotemporal and programmable thermo-haptic stimuli [16,17,18,19,20] is particularly advantageous for enhancing the immersion of VR/AR experiences, including the remote delivery and reproduction of artificial sensations. Recent efforts indicate that the stimulation range of thermal actuators extends beyond the fingers, wrists, and ankles to other body region [21,22,23,24,25,26,27,28,29,30,31]. However, significant engineering challenges remain in scaling the unit actuators for full–body sensations, primarily due to energy efficiency constraints in heating and cooling operations, the relatively higher weight of thermal actuators, and the lower thermal sensitivity of the skin compared to vibrotactile sensitivity. In addition, design considerations should account for thermal sensitivity and discriminability across different body areas [32]. Consequently, stimulation factors—such as the area of stimulation, magnitude and rate of temperature change, and duration of stimulation [33,34,35,36,37]—should be adjusted accordingly for different regions of the body.

**Scheme 1:**
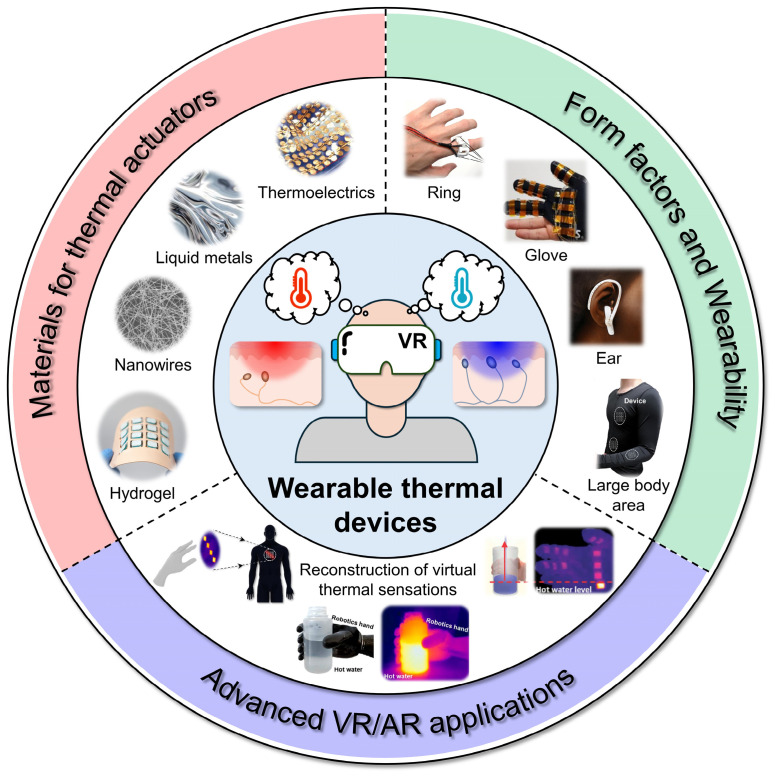
Overview of this review. Reproduced from [19,20,21,29,31,38] with permission from Wiley–VCH. Copyright (2020), the American Chemical Society. Copyright (2015), the National Academy of Sciences. Copyright (2023, 2024), the Elsevier. Copyright (2019), the Royal Society of Chemistry. Copyright (2020).

This review highlights recent advances in wearable thermal devices (WTDs) designed to reproduce artificial thermal sensations for VR/AR applications (Scheme 1). It begins by examining key thermal stimulation factors, including stimulation area, magnitude, and duration, with a focus on thermal sensation mechanisms, perception, and thermoreceptor distribution in the skin through skin–integrated thermal systems. The importance of energy efficiency is also addressed, particularly regarding power requirements for modulating skin temperature based on theoretical modeling. A subsequent section summarizes material choices for fabricating wearable thermal actuators, including metal nanowires, carbon nanotubes, liquid metals, thermoelectric devices, and passive cooling elements. The discussion emphasizes functionalities, device design, operation modes, fabrication processes, and the electrical and mechanical properties relevant to wearable applications. Select advanced examples illustrate how these thermal devices can be integrated into flexible, thin form factors to enable immersive VR/AR experiences through spatiotemporal, programmable thermal stimulation. A concluding section outlines key challenges and future opportunities for skin-integrated thermal devices in advancing next–generation VR/AR systems.

## 2. Thermal Stimulation and Perception in the Skin

When thermal stimulation is delivered from skin-integrated systems to the skin, thermal perception occurs through the activation of cutaneous thermoreceptors, allowing the sensation of temperature. There are two primary types of thermoreceptors: warm thermoreceptors, which respond to thermal stimulation between 36 °C and 45 °C, and cold thermoreceptors, which are activated below 30 °C [39]. If skin temperature rises above 45 °C or falls below 15 °C, nociceptors are triggered, resulting in the sensation of pain [40].

**Figure 1:**
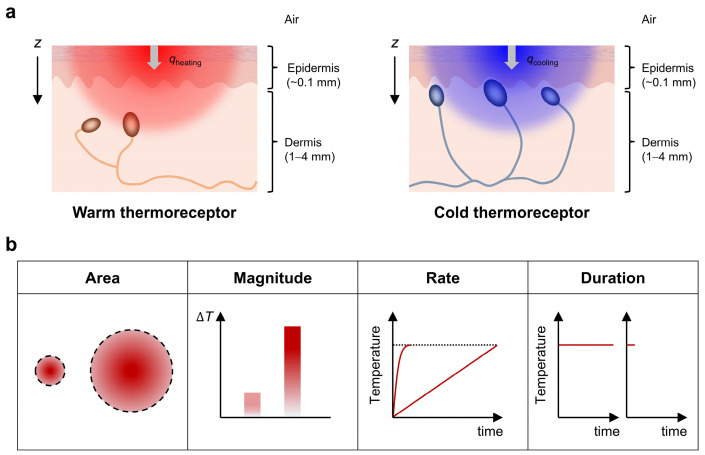
(**a**) Schematic illustration of thermoreceptor distribution across skin layers. (**b**) Key factors influencing thermal stimulation including stimulation area, magnitude and rate of temperature change, and duration of stimulation.

Warm thermoreceptors are mainly distributed in the dermis, whereas cold thermoreceptors are found throughout both the epidermis and dermis, with a higher distribution density than warm thermoreceptors (Figure 1a). The depth and density distribution of both thermoreceptor types are critical for thermal perception, given the relatively low thermal conductivity of the skin [41]. Theoretical calculations using an analytical model of skin temperature [42] provide an estimation example of the power required to exceed warmth and cold perception thresholds:

(1) $$T(x,t)=T_{i}+\frac{2q\sqrt{\frac{\alpha t}{\pi }}}{k}exp\left(\frac{{-x}^{2}}{4\alpha t}\right)-\frac{qx}{k}\left(1-erf\left(\frac{x}{2\sqrt{\alpha t}}\right)\right)$$

where *T*(*x*, *t*) indicates the skin temperature depending on the skin depth (*x*) and the duration (*t*). *T_i_* is the initial temperature of the skin, *q* is the constant heat flux, *α* is the thermal diffusivity of the skin, and *k* is the thermal conductivity of the skin. If we assume *T_i_* = 32 °C, *x* = 0.5 mm for warm thermoreceptors and *x* = 0.2 mm for cold thermoreceptors, then the *q* can be determined by setting the *T* = 40 °C for heating and *T* = 26 °C for cooling, achieved through a constant heat flux. Calculation results indicate that it takes approximately 17 s to reach 40 °C from 32 °C with *q* = 484 W·m^−2^, and 7 s to reach 26 °C from 32 °C with the *q* at the same level. Although target temperatures can be reached by applying a lower heat flux, a low rate of temperature change (i.e., prolonged stimulation) can lead to thermal adaptation, potentially disrupting the temperature sensing function of the skin.

Since the human body exhibits varying thermal sensitivities across different regions [32], stimulation magnitude should be controlled in a relative manner. The discriminability of thermal stimulation depends on several factors, including the stimulation area, the magnitude of temperature change from the baseline skin temperature, the rate of temperature change, and the duration of stimulation (Figure 1b). The literature indicates that spatial discrimination for thermal stimulation is lower than for tactile stimulation [34,35]. Additionally, spatial summation explains the thermal sensitivity of the skin, as the perceived thermal sensation—whether from conducted or radiant heat—increases with the size of the stimulation area [36,37]. The steeper the stimulation rate, the lower the threshold for perception [43], indicating that rapid temperature changes are more readily detected by thermoreceptors compared to gradual changes. Shorter–lasting stimulation using laser pulses of equal energy raises the skin temperature more rapidly, resulting in higher-amplitude brain signals [44]. Moreover, skin type (glabrous vs. hairy) should also be considered for the thermal perception [45], as differences in skin properties may influence thermal sensitivity across individuals. This suggests that, for improved thermal perception, patterned stimulation with independent, programmable control is desirable.

## 3. Wearable Thermal Devices (WTDs)

### 3.1. Resistive Heating

Resistive heating, also well known as Joule heating, is the process by which electrical energy is converted into heat energy when an electric current flows through a conductor. Described by Joule’s law of heating, the heat generated (*Q*) is proportional to the square of the current (*I*^2^), the resistance of the conductor (*R*), and the time (*t*) for which the current flows:

(2) $$Q=I^{2}Rt$$

The mechanism of resistive heating involves the movement of free electrons through a conductor, where collisions with atoms transfer kinetic energy, increasing atomic vibrations and thus generating heat. Resistive heating offers a straightforward approach for constructing skin-integrated thermal interfaces, as the temperature can be easily controlled by applying voltage or current to the fabricated device.

Recently emerging nanomaterials, including metallic nanomaterials (e.g., metal nanowires and carbon nanotubes), liquid metals, and conductive polymers, can be readily incorporated into deformable devices, replacing traditional rigid materials such as bulk metal conductors and metal oxides. These nanomaterials serve as the foundation for developing wearable thermal devices (WTDs) with thin, flexible, and stretchable format. The fabrication of WTDs using these materials is typically achieved by incorporating them into insulating elastomers, cotton, and fabrics. Here, we introduce key materials and fabrication strategies for harnessing Joule heating in recently developed WTDs. The materials and structure, temperature range, required electrical input, and form factors of the WTDs utilizing the resistive heating mechanism are summarized in Table 1.

#### 3.1.1. Silver Nanowires (Ag NWs)

The advantages of silver nanowires (Ag NWs) include their excellent conductivity [65,66,67,68], flexibility [51,69,70,71,72,73], transmittance [52,66,74,75,76,77,78], and solution processibility [38,79,80,81] making them highly suitable for use in WTDs.

**Figure 2:**
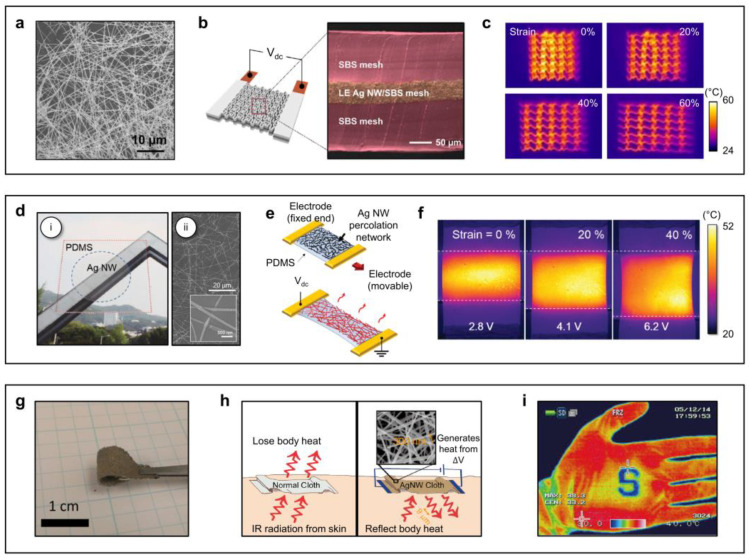
Silver nanowires (Ag NWs)–based WTDs. (**a**–**c**) SEM image of Ag NWs (**a**); schematic illustration of the mesh heater, comprising a heating layer of Ag NWs and SBS (left), and SEM image showing the cross–sectional view of the mesh heater (right) (**b**); and IR images of the mesh heater under applied strains of 0, 20, 40, and 60% with a constant applied voltage of 0.75 V (**c**). Reproduced from [38] with permission from the American Chemical Society. Copyright (2015). (**d**–**f**) Photograph (i) and SEM image (ii) of the Ag NWs/PDMS electrode (**d**); schematic illustration of the stretchable and transparent heater composed of Ag NWs percolation network on a PDMS film (**e**); and IR images of the Ag NWs/PDMS heater under various strains with an adjust voltage to maintain a constant temperature (50 °C) (**f**). Reproduced from [52] with permission from Wiley–VCH. Copyright (2015) (**g**–**i**) Photograph of Ag NWs–cloth (**g**); conceptual illustration of the NWs cloth with thermal radiation insulation and active heating (**h**); and IR image of the Ag NWs–cloth cut into an S shape and placed in the palm, demonstrating its ability to block human–radiated IR and displaying cold color (**i**). Reproduced from [51] with permission from the American Chemical Society. Copyright (2015).

The first example is a stretchable heater using nanocomposite of ligand–exchanged Ag NWs for wearable articular thermotherapy [38]. In this example, a soft and stretchable heating element that is lightweight and thin and is conformally integrated with the human joints and the skin for effective heat transfer and thermotherapy. The heater is composed of highly conductive Ag NWs/elastomer nanocomposite. The ligand exchange (LE) of Ag NWs allows the nanowires to be homogeneously dispersed in the elastomeric media. This excellent homogeneity leads to mechanically and electrically uniform characteristics and processability of the composite into various patterns, which enables reliable, large–area heating over the entire joint surfaces. Figure 2a shows a scanning electron microscope (SEM) image of LE Ag NWs. The average diameter and length are ~150 nm and ~30 μm, respectively. Figure 2b shows a schematic illustration of the fabricated mesh heater, which operates upon the direct current (DC) voltage applied. The heater consists of encapsulation layers (illustrated with a white color encapsulation; the material is styrene–butadiene–styrene; SBS) and a heating layer (LE Ag NW/SBS). Thermal welding with heat and pressure was used to combine and bond each layer. The cross–sectional SEM image in Figure 2b shows the welded interfaces. Figure 2c shows the infrared (IR) camera images of meshes at applied strains of 0, 20, 40, and 60% and applied voltage of 0.75 V, indicating the high stretchability and operational stability of the mesh heater upon the deformation. A low system modulus of the mesh heater allows the wrist wearability and uniform and stable heating despite large joint movements.

Another example is a stretchable and transparent Ag NW (STAN) heater that exploits the Ag NW percolation network in a polydimethylsiloxane (PDMS) [52]. Figure 2d shows a digital image of as–prepared STAN electrode. The area with Ag NW percolation network is denoted with a blue dotted line. The SEM image on the right in Figure 2d shows individual Ag NWs with an inset showing a magnified image at nanowire junction. Figure 2e illustrates the scheme of the STAN electrode. Uniformly distributed Ag NWs at random directions forming a percolation network on a PDMS film, which is prepared by vacuum filtration transfer. A constant DC bias voltage is applied at two ends of the Ag NWs network electrode to induce electrically driven resistive Joule heating. The STAN heater can be stretched during its heating operation since the PDMS, the underlying substrate as well as the Ag NWs network, the conducting medium are both stretchable while maintaining their electrical conductance and optical transmittance. Figure 2f shows the IR images of the STAN heater under applied strains of 0, 20, and 40% with adjusted voltage to maintain constant temperature (50 °C). Reducing the thickness of the PDMS film (<500 μm) enables the faster response time of the STAN heater (≈10 s), which is considerably faster than the Ag NWs–based film heater on ultrathin glass substrate and even comparable to gold (Au) patterned microheater, owing to efficient heat transfer to the adjacent elastomer and low volumetric heat capacitance of PDMS.

The nanowire–coated textile can also be efficiently explored as a heat saver from human bodies [51]. Figure 2g shows a digital image of Ag NWs–cloth showing flexibility. Figure 2h illustrates an effective method of personal thermal management with a metallic nanowire textile. Normal cloth traps the air surrounding the human body to decrease heat transfer via convection and conduction, but its high emissivity (0.75–0.9) provides little radiative insulation. After coating the cloth with metallic nanowires and forming a conducting network, the majority of human body radiation is reflected back toward the body, greatly enhancing the insulation performance. The spacing between nanowires is controlled to be between 200 and 300 nm. Human body radiation is generally 9 μm in wavelength, so most of it ‘sees’ the nanowire cloth as a continuous metal thin film and is reflected. However, unlike a continuous metal film, which is vapor impermeable, the spacing of the nanowire network is much larger than a water molecule. Therefore, water vapor generated by perspiration can easily escape, making the user feel comfortable. In addition to reflecting body radiation, if an electricity source is connected to the textile, the low sheet resistance of the nanowire network can provide effective Joule heating to further increase the skin temperature. Figure 2i shows IR image of Ag NW–cloth. The cloth was cut into an S shape and placed in the palm. The Ag NWs–cloth was able to block the human–radiated IR and shows the cold color, due to the low emissivity and the low thermal radiation of Ag NWs coating.

#### 3.1.2. Copper Nanowires (Cu NWs)

Copper nanowires (Cu NWs) present a promising alternative for resistive heaters, offering abundance [82,83] and sustainability [84] that enhance their potential for scalable [85,86] and eco–friendly production [87,88]. Although copper has slightly lower electrical conductivity than silver, it facilitates effective and uniform heat generation at a significantly reduced cost [31,56,62]. The mechanical flexibility, scalability, and economic viability of Cu NWs position them as strong candidates for high–performance and cost–effective WTDs [89,90,91,92].

**Figure 3:**
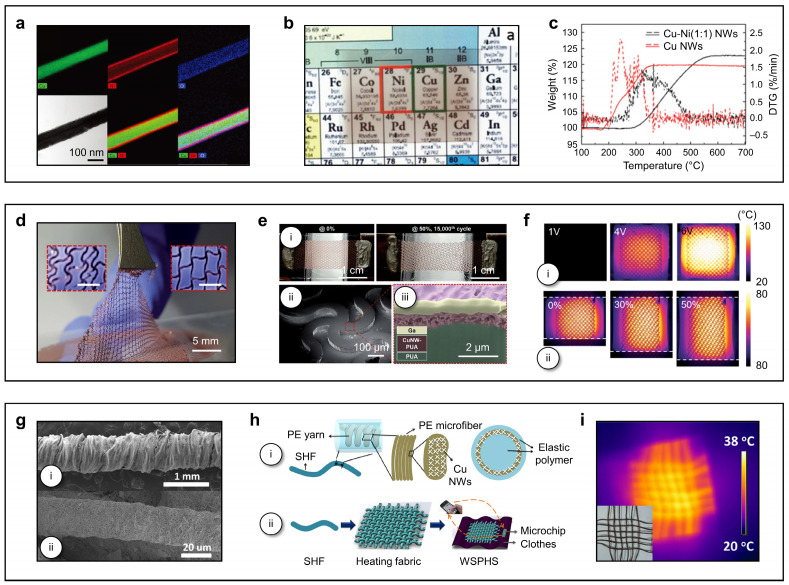
Copper nanowires (Cu NWs)–based WTDs. (**a**–**c**) TEM image and elemental maps of Cu–Ni NWs (**a**); photograph of the Cu–Ni (1:1) transparent conductive film with sheet resistance (*R*_s_) of 300 Ω sq^−1^ and transmittance (*T*) of 76% (**b**); and thermogravimetry (dashed line) and differential thermogravimetry (solid line) curves of neat Cu NWs and Cu–Ni NWs (**c**). Reproduced from [56] with permission from the Royal Society of Chemistry. Copyright (2015). (**d**–**f**) Photograph of the patterned Cu NWs–PUA electrode with a tweezer, with inset images showing the meandering shape and the structurally expanded shape when stretched (scale bar = 1 mm) (**d**); photograph showing the deformations in the electrode after the 50% stretching test was repeated 15,000 times (**i**), SEM image (**ii**) and cross–section SEM image (**iii**) of the patterned Cu NWs–PUA electrode (**e**); and IR images of the patterned Cu NWs–PUA heater in response to increasing voltage (**i**), and when stretched during heating (**ii**) (**f**). Reproduced from [31] with permission from the Royal Society of Chemistry. Copyright (2020). (**g**–**i**) SEM images of PE yarn coated with Cu NWs (**i**) and a localized single PE microfiber coated with Cu NWs (**ii**) (**g**); schematic illustration of the hierarchical structure of the SHFs (**i**), and integration of SHFs into WSPHS for wearable applications (**ii**) (**h**); and IR image of the heating fabric under DC voltage of 1.8 V, with an inset displaying a photograph of the heating fabric (12 SHFs woven in a cross pattern) (**i**). Reproduced from [62] with permission from the American Chemical Society. Copyright (2016).

In order to improve the oxidation resistance of Cu NWs, a one–pot synthesis of copper–nickel (Cu–Ni) core–shell nanowires with a tunable Ni–shell thickness has been proposed [56]. Figure 3a shows a transmission electron microscope (TEM) image and elemental maps of Cu–Ni NWs with a Cu/Ni molar ratio of 1:1. Figure 3b shows a digital image of a transparent conductive film (TCF) that is fabricated by depositing the Cu–Ni NWs ink onto the poly(ethylene terephthalate) (PET) substrate by spray coating. Optimal conductivity and oxidation resistance of the Cu–Ni NWs was achieved with a 10 nm thick Ni shell. Thermogravimetric analysis (TGA) indicates that the oxidation onset temperature is increased from 180 °C for neat Cu NWs to 270 °C for Cu–Ni NWs (Figure 3c).

Laser-assisted dual-function Cu NWs polyurethane acrylate (PUA) patterns serve as feedback controllable stretchable heaters [31]. Figure 3d shows a digital image of patterned Cu NWs–PUA electrode with a tweezer. The pattern was formed by cutting the 25 μm thick Cu foil into a serpentine shape using the UV laser beam (100–150 mW). The results of repeated tensile tests of 15,000 times indicate that the resistance growth rate approaches a constant value at strains up to 50% (Figure 3e(i)). However, for strains exceeding 50%, the resistance continues to increase with the number of cycles. The microstructure of the patterned Cu NWs–PUA electrode is shown in the SEM image (Figure 3e(ii)) and the cross–sectional SEM image (Figure 3e(iii)). A gallium rod is placed on the cutting boundary to prevent the electrode from being moved and distorted during the cutting process using a focused ion beam (FIB). Figure 3f shows IR images of the heater at applied voltages of 1, 4, and 6 V, indicating the stable temperature distribution when stretched at strains of 0, 30, and 50%.

Composite fiber with Cu NWs provides another type of high performance stretchable heating fibers (SHFs) due to stable electrical conductivity under various deformations such as bending, twisting, and stretching [62]. Two SEM images show polyester (PE) yarn coated with Cu NWs (Figure 3g(i)) and a localized single PE microfiber coated with Cu NWs (Figure 3g(ii)). The entire fabrication process of a hierarchical structured Cu NWs–PE based SHFs and a subsequent wearable and smart personal heating system (WSPHS) is shown in Figure 3h. The Cu NWs were initially deposited onto a helical yarn composed of PE microfibers. Hydrogen plasma treatment was then applied to enhance the electrical conductivity of the Cu NWs network. Subsequently, the structure was dip–coated in liquid silicone rubber and cured to form a protective sealing layer. The resulting SHFs were woven into a heating fabric, combining excellent durability under various deformations. For a WSPHS, the SHFs were integrated into the heating fabric, alongside a microcontroller unit (MCU) embedded in clothing, enabling interaction with a smartphone. Figure 3i shows IR image of heating fabric under DC voltage of 1.8 V and a digital image of heating fabric (12 SHFs woven in a cross pattern).

#### 3.1.3. Carbon Nanotubes (CNTs)

The exceptional mechanical flexibility [93,94,95], stretchability [47,60,96], and tensile strength [97,98] of carbon nanotubes (CNTs) make them ideal for resistive heaters that require frequent bending or deformation, despite lower electrical conductivity compared to metallic nanowires. Notably, unlike Cu NWs, CNTs exhibit high resistance to oxidation, ensuring long–term reliability and chemical stability [99,100]. Additionally, CNTs can be easily integrated into textiles and polymers using scalable techniques such as spray coating [60,63,101] or inkjet printing [102,103]. Their biocompatibility [104,105] and potential for chemical modification [106] further enable versatile and customizable applications in WTDs.

**Figure 4:**
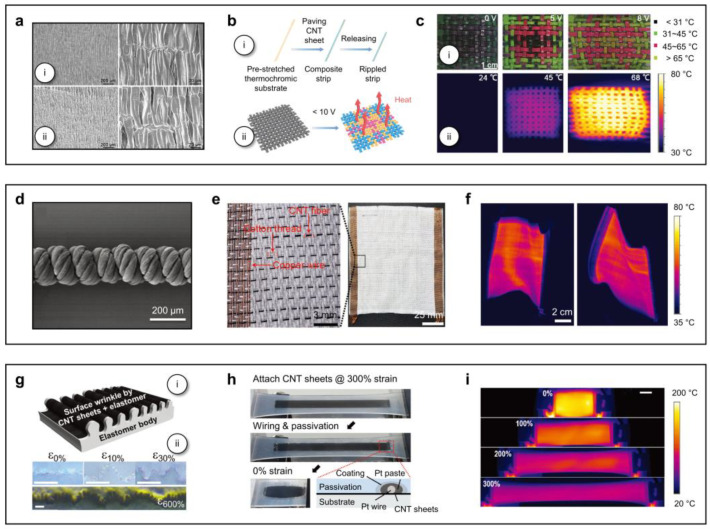
Carbon nanotubes (CNTs)–based WTDs. (**a**–**c**) SEM images of a CNT electrode at low (left) and high (right) magnifications before stretching (**i**) and after stretching (**ii**) (**a**); schematic illustration of the fabrication of the stretchable strip–shaped TRH (**i**), and the resulting smart TRH textile operating under an applied voltage below 10 V (**ii**) (**b**); and photographs (**i**) of the TRH textile under applied voltage of 0 and 5 V, and after stretching by 40% at a voltage of 8 V (**i**), and corresponding IR images (**ii**) (**c**). Reproduced from [60] with permission from the Royal Society of Chemistry. Copyright (2017). (**d**–**f**) SEM image of HHF (**d**); photograph of the heating textile made of CNT–based fiber materials at high (left) and low (right) magnifications (**e**); and IR images of the heating textile under distortion at an applied voltage of 9 V (**f**). Reproduced from [63] with permission from Wiley–VCH. Copyright (2018). (**g**–**i**) Schematic illustration of a surface–wrinkled CNT sheet on an elastomer heater (**i**), and optical microscope images for cross–sections of samples with different pre–stretching ratios from 0 to 600% (**ii**) (**g**); photographs showing the fabrication process of a stretchable heater from stacked CNT sheets on a pre–strained elastomer (**h**); and IR images of the stretchable heater at different strains ranging from 0 to 300%, operating under a constant voltage of 34 V (**i**). Reproduced from [47] with permission from Wiley–VCH. Copyright (2018).

A flexible and stretchable strip–shaped thermochromic resistive heater (TRH) was fabricated by incorporating an aligned CNT sheet and a thermochromic silicon elastomer [60]. SEM images of a CNT electrode at low and high magnifications before stretching (Figure 4a(i)), and after stretching (Figure 4a(ii)), respectively. The wrinkle morphology of the CNT layer allows for high stability in electrical resistance. A thermochromic silicone elastomer, prepared by spin–coating a mixture of silicone elastomer precursor and thermochromic microcapsule ink, is pre–stretched (Figure 4b(i)). Aligned CNT sheets, drawn from a spinnable CNT array synthesized via chemical vapor deposition (CVD), are then applied to the stretched substrate. Upon releasing the substrate, a stretchable, strip–shaped TRH is formed. The resulting smart heater textile, woven from these TRHs, can visually display real–time temperature patterns at a safe human–operable voltage (<10 V) (Figure 4b(ii)). Figure 4c displays digital images of the TRH textile under stepwise voltage increases of 0, 5, and 8 V, demonstrating its thermochromic response (Figure 4c(i)). At 8 V, the textile is stretched by 40%. Corresponding IR images illustrate the temperature distribution (Figure 4c(ii)). This smart visual display allows users to easily and effectively assess the temperature range of the TRH textile based on its pattern.

Designing a hierarchically helical structure with aligned CNT fibers as the fundamental unit served as an energy–efficient heating fiber [63]. A SEM image in Figure 4d shows microstructure of the hierarchically helical fiber (HHF). The HHF was fabricated by twisting CNT ribbons synthesized via floating catalyst CVD. The CNTs, with diameters around 20 nm, were aligned along the ribbon length, creating nano–scaled voids. These HHFs were then woven with cotton threads and copper wires to form heating textiles. The cotton threads acted as supporting substrates, while the copper wires served as conductors, eliminating the need for additional conductive paste or tape. This method facilitated large–scale fabrication and improved washability (Figure 4e). Figure 4f shows IR images of the heating textile under distortion at 9 V, demonstrating stable performance even under deformation.

An extremely stretchable Joule heater was created using CNT sheets on pre–strained elastomer [47]. Figure 4g shows the surface–wrinkled CNT sheet on elastomer (Figure 4g(i)) and optical images for cross–sections of samples at various pre–stretching ratios (0, 10, 30, and 600%) (Figure 4g(ii)). Figure 4h illustrates the fabrication process, involving CNT sheets stacked on a pre–strained elastomer with connecting electrical leads. The multiwalled CNT sheets, serving as ultra–flexible conductive layers, form folds through surface buckling. When stretched, the elastomeric polymers cannot return to their original shape as the CNT layer cannot shrink. Figure 4i shows IR images of the heater at strains of 0, 100, 200, and 300%, with temperature reduction at high strains due to decreased electrical power density as sample length increases.

#### 3.1.4. Liquid Metals (LMs)

Liquid metals (LMs), such as gallium and its alloys such as eutectic–gallium–indium (EGaIn), and gallium–indium–tin (GaInSn; “Galinstan”) offer unique advantages for WTDs. Their fluidic nature allows conformation to irregular surfaces and flexibility under stretching or bending without compromising functionality [53,64,107,108]. Additionally, LMs exhibit self–healing properties, reconfiguring after deformation or damage to maintain consistent electrical pathways and enhance durability [109]. Scalable methods like printing and microfluidic channels enable precise patterning on various substrates, while polymer encapsulation prevents leakage and improves durability [110,111].

**Figure 5:**
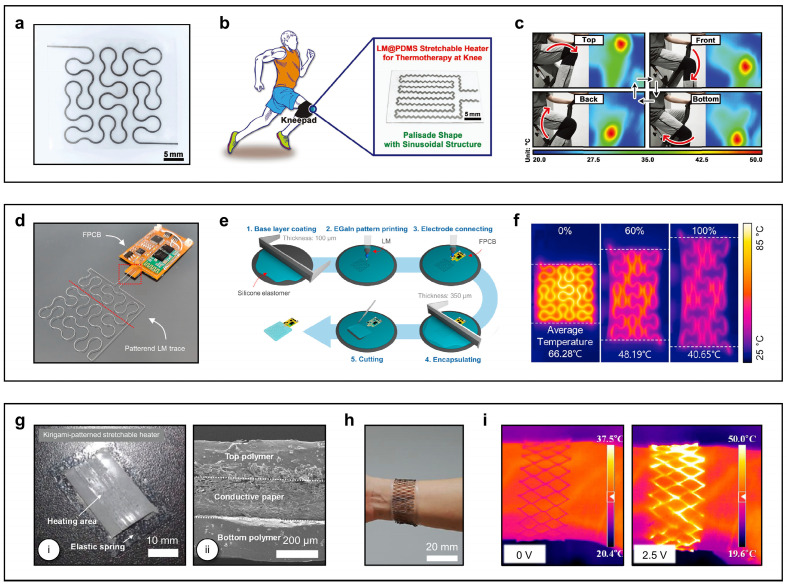
Liquid metals (LMs)–based WTDs. (**a**–**c**) Photograph of LM@PDMS stretchable heater with fractal structure (**a**); schematic illustration of the working condition of LM@PDMS heater and a photograph of this heater with a palisade shape and sinusoidal structure, designed for thermotherapy on the knee (**b**); and photographs and corresponding IR images of exercise at different states, where the kneepad worn by the user is embedded with the LM@PDMS heater (**c**). Reproduced from [53] with permission from Wiley–VCH. Copyright (2019). (**d**–**f**) Photograph of the patterned LM trace after electrode connection (**d**); schematic illustration of the manufacturing process for the wearable LMs–based heater (**e**); and IR images of the fractal-shaped LMs–based heater under increasing strain during heating (**f**). Reproduced from [64] with permission from the Elsevier. Copyright (2023). (**g**–**i**) Photograph (**i**), and cross–sectional SEM image (**ii**) of the kirigami–patterned stretchable heater (**g**); photograph of the band–type kirigami heater attached to the wrist (**h**); and IR images of the kirigami heater attached to the wrist before (left) and after (right) applying voltage (**i**). Reproduced from [49] with permission from the American Chemical Society. Copyright (2017).

A printable LM–based stretchable heater was proposed for wearable thermotherapy [53]. Figure 5a displays a composite heater comprising patterned LMs and PDMS (LM@PDMS), fabricated via direct ink writing (DIW). A mushy mixture of LMs and uncured PDMS is patterned onto a PDMS film using air injection, cured at 60 °C for 30 min, and encapsulated with PDMS to form the stretchable heater after mold removal. The fractal structure enables >100% stretchability, making it suitable for thermotherapy on the knee joint (Figure 5b). The heater, designed with a sinusoidal palisade pattern for uniaxial knee strain, is embedded in a kneepad. IR images confirm consistent heating performance during exercise, demonstrating its potential for wearable thermotherapy (Figure 5c).

A recent study explored a simplified circuit control system for LMs-based wearable heaters without complex structures or deep learning [64]. Figure 5d shows a digital image of the patterned LMs trace after electrode connection. The manufacturing process (Figure 5e) involves coating a 100 µm silicone layer on a silicon wafer, patterning LMs using DIW, and attaching a custom flexible printed circuit board (FPCB) to align with the LMs trace. Figure 5f presents IR images of a Peano curve design under 0, 60, and 100% strain during heating. At a constant voltage, increased strain enlarges the area and reduces resistance, causing the average temperature to drop from 66.28 to 40.65 °C. This study demonstrates the feasibility of supplementing heat generation through circuit control, with a predictable relationship between strain, area, and resistance.

Another method for producing stretchable heaters involves a simple kirigami–patterning approach using conductive paper [49]. Figure 5g shows a digital image of the fabricated kirigami–patterned heater, made from aluminum (Al) paper sandwiched between thin elastomeric polymers via a single–step cutting process after elastomer spin coating. The heating area measures ~33 × 10 mm^2^, achieving >40 °C at 1.2 V with a rapid thermal response (<60 s). The kirigami design enables extreme stretchability (>400%) while maintaining stable performance, with the heater nearly fully recovering its original shape due to the elastic springs’ restoring force (Figure 5h). IR images in Figure 5i demonstrate the heater on a wrist joint, showing no heating at 0 V and activation at 2.5 V.

### 3.2. Thermoelectrics

Thermoelectric devices (TEDs) operate based on the Peltier effect, directly converting electrical energy into thermal energy to either lower or raise temperature [112]. Compared to Joule heating, a key advantage of TEDs is their ability to provide active cooling. Additionally, their bidirectional functionality, enabling both cooling and power generation, along with a fast response time offers further benefits for designing multimodal WTDs.

In TEDs, heat is carried by the electric current. The device structure comprises a semiconductor material (*p*– or *n*–type) sandwiched between metal conductors. When a voltage is applied, electrons travel from one metal layer through the semiconductor to the opposite metal layer. To transition from the metal into the semiconductor, electrons must overcome an energy barrier corresponding to the difference between the Fermi level (*E*_F_) of the metal and the conduction band minimum of the semiconductor. Only “hot electrons” with sufficient energy can cross this barrier, causing the metal on the left side of the structure to cool as high–energy electrons leave. By reversing the direction of the current, the heating and cooling effects are inverted. TEDs can also convert heat into electrical power. In this mode, a heat source applied to the hot side of the structure excites electrons, enabling them to escape into the semiconductor and travel to the opposite side, where they release their energy. This energy release generates an electric current, effectively converting thermal energy into electrical energy.

In general, good thermoelectric materials require high electrical conductivity (*σ*) to efficiently transfer heat-bearing electrons while minimizing Joule heating. They also require low thermal conductivity (*k*) to reduce heat loss from electrons as they traverse the sandwich structure. The dimensionless figure-of-merit for thermoelectric materials expressed as *ZT*:

(3) $$ZT=\frac{S^{2}\sigma T}{k}$$

where *S* is the Seebeck coefficient (in units of V/K or V/°C), representing the voltage induced by a temperature gradient along the material. *σ* and *k* represent the electrical conductivity and thermal conductivity of the material, respectively. These three parameters (*S*, *σ*, and *k*) are interdependent, influenced by factors such as the band structure, carrier concentration, and other material properties [113]. Numerous studies have focused on enhancing the *ZT* value of thermoelectric materials. Classic examples of thermoelectric materials that operate at room temperature (300 K), such as bismuth telluride (Bi_2_Te_3_) and lead telluride (PbTe), have a *ZT* value of approximately 1 [113,114].

Here, we present key studies that explore engineering of thermoelectric materials and design strategies for WTDs in VR/AR applications. The exemplary thermoelectric materials focus on Bi_2_Te_3_–based alloys, which exhibit stable and excellent performance. The materials and structure, temperature range, required electrical input, and form factors of the WTDs utilizing the Peltier effect are summarized in Table 2.

**Figure 6:**
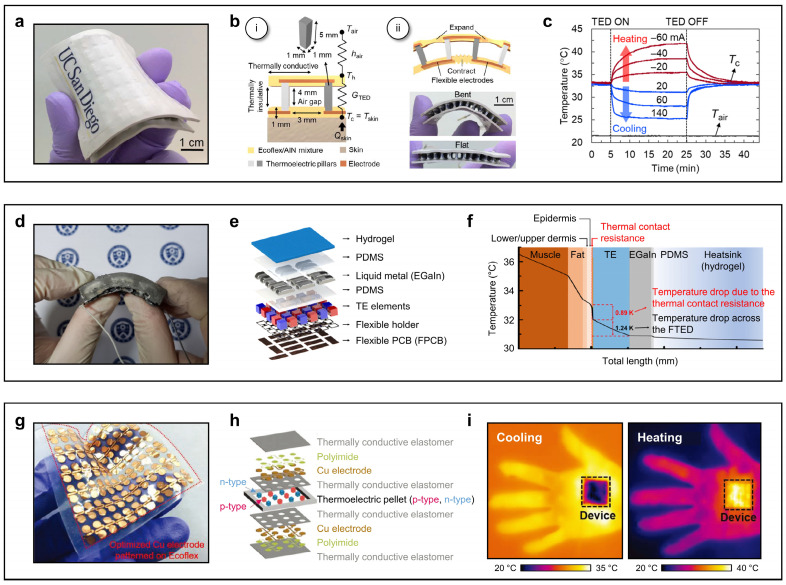
Thermoelectric–based WTDs. (**a**–**c**) Photograph of the flexible thermoelectric device (TED) with dimensions of 5 × 5 cm^2^ (**a**); schematic illustration depicting the TED design (**i**) and a diagram with corresponding photographs demonstrating its flexibility (**ii**). During bending, the top stretchable sheet undergoes expansion, while the bottom stretchable sheet contracts (**b**); and temperature profile of the TED under cooling and heating operation (**c**). Reproduced from [30] with permission from the American Association for the Advancement of Science. Copyright (2019). (**d**–**f**) Photograph of the assembled flexible TED (**d**); exploded view schematic illustration detailing each component of the flexible TED (**e**); and temperature profile illustrating heat transfer from the human body to the flexible TED (**f**). Reproduced from [116] with permission from the Elsevier. Copyright (2019). (**g**–**i**) Photograph of the serpentine Cu electrode mounted on Ecoflex (**g**); exploded view schematic illustration of the stretchable TED–based device (**h**); and IR images of a 1 mm thick hand–shaped PDMS with the TED–based device positioned beneath it, showing cooling (left) and heating (right) operations (**i**). Reproduced from [29] with permission from Wiley–VCH. Copyright (2020).

An innovative concept of double elastomer layer design embedding an air gap insulation layer between two stretchable sheets and high–*ZT* inorganic thermoelectric pillars with optimized aspect ratio and spatial density [30]. It demonstrates a large cooling effect of more than 10 °C to the skin without the use of any heat sinks. Figure 6a shows a digital image of this flexible TED. To achieve mechanical flexibility and high cooling performance, a double elastomer layer design was implemented, sandwiching rigid thermoelectric pillars (1 × 1 mm^2^ cross-section, 5 mm height, 3 mm gap) between two 1 mm thick stretchable Ecoflex sheets separated by a 4 mm air gap (Figure 6b(i)). This design ensures flexibility despite the rigidity and high aspect ratio of the thermoelectric pillars, significantly enhancing cooling performance. As shown in Figure 6b(ii), bending the TED causes the top sheet to expand and the bottom sheet to contract, a common feature in flexible TEDs. Figure 6c presents the time–dependent cooling and heating effects (*T*_c_) at the bottom of the TEDs under applied currents ranging from −60 to 140 mA. *T*_c_ responded instantly to the current and stabilized at a plateau within minutes.

Another example demonstrates the bending capability of TEDs utilizing liquid–metal electrodes encapsulated in PDMS above the bending neutral axis and FPCB electrodes below, leveraging the stretchability of the LMs and the flatness of the FPCB to minimize thermal contact resistance [116]. Figure 6d shows a bent TED with its structure detailed in Figure 6e. The temperature profile from muscle to heatsink in Figure 6f highlights that maximizing power output from body heat harvesting requires optimizing the temperature drop across the thermoelectric materials under the given skin and ambient temperatures. The TEDs can achieve skin–conformal, flexible designs by replacing metal plates with thin metal traces for heat conduction. The Cu serpentine interconnection electrode design enhances stretchability and deformation resistance while maintaining electrical conductivity [29]. Figure 6g shows a serpentine Cu electrode on Ecoflex, with the complete TED structure detailed in Figure 6h. Figure 6i demonstrates localized cooling and heating effects of these 2.7 × 2.7 cm^2^ flexible TEDs beneath a 1 mm thick hand–shaped PDMS layer.

### 3.3. Integrating Passive Cooling Mechanism

In wearable devices, emerging passive cooling is crucial for managing heat dissipation, ensuring user comfort, device performance, and lifetime without relying on power–intensive or bulky cooling mechanisms. It leverages natural processes like evaporation, conduction, convection, and radiation to dissipate heat effectively. Materials such as lightweight thermal insulators (e.g., aerogels) [118,119,120], phase change materials (PCMs) [121,122] that absorb and release heat, high–emissivity coatings for radiative cooling [123,124,125], and porous or evaporative materials (e.g., hydrogels) [126,127,128,129] can be used. Passive cooling offers several advantages for wearables, including energy efficiency, extended battery life, lightweight and compact integration, and silent operation. By preventing heat buildup near the skin, it ensures user safety and comfort, while its environmentally friendly and reliable nature aligns with sustainable and durable design principles. Furthermore, passive cooling reduces manufacturing complexity and costs, making it a critical strategy for improving the functionality and usability of wearable technology.

**Figure 7:**
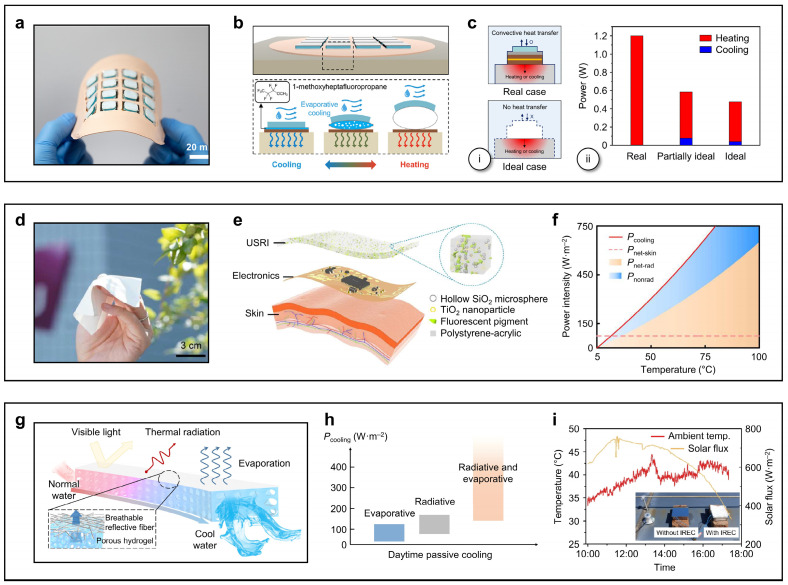
Passive cooling–based WTDs. (**a**–**c**) Photograph of a thin, flexible, and wireless system designed for generating programmable patterns of thermal sensation across large areas of the skin (**a**); schematic illustration depicting the working principle of the switchable thermal barrier (STB) at the skin interface (**b**); and side view model of a thermal module for heating and cooling under real and ideal cases (**i**), the power consumption for heating a skin phantom from 27 °C to 40 °C within 7 s (red bar) and cooling from 40 °C to 32 °C within 60 s (blue bar) across ideal, partially ideal, and real cases (**ii**) (**c**). Reproduced from [19] with permission from the National Academy of Sciences. Copyright (2023). (**d**–**f**) Photograph of an ultrathin, soft, radiative cooling interfaces (USRI) layer (**d**); exploded view schematic illustration detailing the components and assembly method of the USRI (**e**); and a comparative analysis of cooling power from radiative and nonradiative processes in wearable devices as a function of the above–ambient temperature induced by Joule heating (**f**). Reproduced from [18] with permission from the American Association for the Advancement of Science. Copyright (2023). (**g**–**i**) Schematic illustration of the hydrogel–based integrated radiative and evaporative cooling (IREC) system (**g**); net cooling power range for evaporative, radiative, and IREC system during daytime operation (**h**); and measured solar flux and air temperature during an outdoor cooling test (**i**). Reproduced from [126] with permission from the Tsinghua University Press.

Evaporative cooling, thermally switchable interfaces, and wireless stretchable electronics form the foundational technologies enabling power–efficient, programmable thermal stimulation across large skin areas with closed-loop control (Figure 7a) [19]. Each thermal module comprises a passive cooling hydrogel, a Cu Joule heater with a Peano fractal shape, and a switchable thermal barrier (STB) in a layered structure. Figure 7b (top) illustrates the STB, consisting of a thin aluminized ethylene vinyl alcohol (EVOH) bladder (12 μm thick) housing 30 μL of low boiling point liquid (1–methoxyheptafluoropropane, boiling point 34 °C). The working principle at the skin interface is depicted in Figure 7b (bottom). Figure 7c highlights simulation benchmarks under ideal case (eliminating parasitic heat loss and actuator thermal mass). Heating the skin by ∆*T* ≈ 13 °C (27 °C to 40 °C) within 7 s requires ~0.5 W, while cooling by ∆*T* ≈ −8 °C (40 °C to 32 °C) within 60 s requires ~0.04 W. The energy to cycle skin temperature using an optimized module is ~1.2 W, approximately 2.4 times the ideal case (~0.5 W). Reducing thermal module mass could bring this closer to ~1.5 times the ideal case.

Ultrathin, soft, radiative cooling interfaces (USRI) enable advanced thermal management in skin electronics [18]. Figure 7d shows a digital image of the prototype device, and Figure 7e provides an exploded view of its components and assembly. The USRI features a micrometer–thick polymeric coating with near–unit infrared emittance, high solar reflectance, and robust mechanical flexibility. Skin–like electronics coated with this USRI demonstrate significant thermal management improvements, with a maximum temperature reduction of 56 °C. The USRI maintains stable cooling even under extreme deformations such as bending, twisting, folding, and stretching. Figure 7f compares the cooling power of radiative and nonradiative processes in wearable devices as a function of the above-ambient temperature caused by Joule heating.

An integrated radiative and evaporative chiller (IREC) demonstrates synergistic cooling performance [126]. The IREC consists of polyacrylamide hydrogels with a breathable poly(vinylidene fluoride–co–trifluoroethylene) fiber layer, achieving a practical average daytime cooling power of 710 W m^−2^. The hydrogel is polymerized at sub–zero temperatures to create a porous water transfer channel, with a reflective fiber layer on the surface. Through water evaporation, fiber radiation, and visible light reflection, IREC effectively cools the hydrogel during the day (Figure 7g). Net cooling power comparisons in Figure 7h highlight the synergistic effects of radiative and evaporative cooling. Outdoor testing conducted on 14 July 2022, in Beijing, confirmed the cooling performance in containers, with solar flux ranging from 600 to 700 W m^−2^ and air temperatures from 35 to 42 °C (Figure 7i).

## 4. Form Factors and Wearability of WTDs

Recent research activities on the aforementioned WTDs have explored various form factors, including conventional heat–mounted gear [130], finger rings [21], wristbands [22,23,25,30,38,49,58,63], gloves [26,27,31,50,131], knee pads [48,53], fabrics [18,62,126], ear hooks [28], and skin–integrated wearables for covering large body areas [19,20,132]. The term “form factor” refers to the overall configuration of wearable devices, encompassing their physical design, shape, and size. It determines how the devices are structured and worn, significantly influencing their usability, functionality, comfort, and potential for long-term adoption [133].

**Figure 8:**
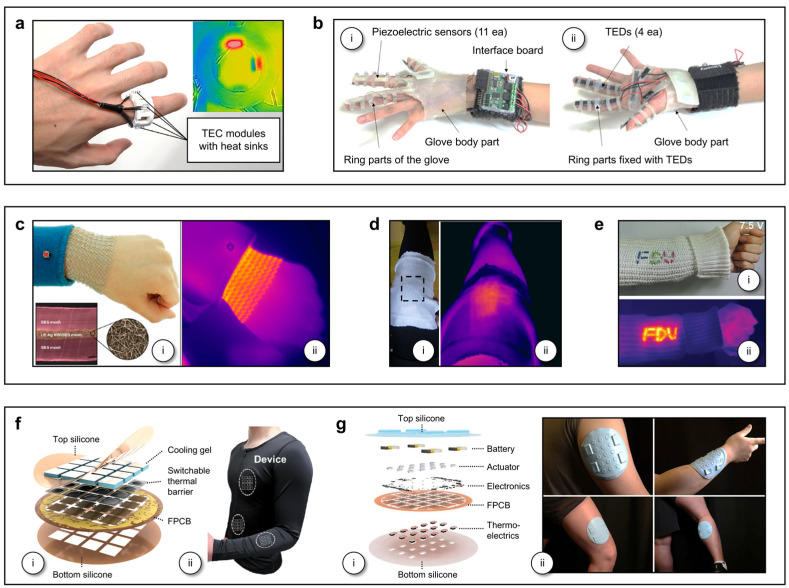
Various form factors of selected WTDs. (**a**) Photograph of the smart ring prototype featuring four thermoelectric modules positioned around the finger. The inset displays an IR image of the ring under stimulation. Reproduced from [21] with permission from the Elsevier. Copyright (2019). (**b**) Photographs of the thermal display glove system for dorsal view (**i**), and palmar view (**ii**). Reproduced from [26] with permission from the Springer Nature. Copyright (2020). (**c**) Photograph of the wrist heater with an SEM image showing the welded interface (**i**), and IR image of the heater while worn on the wrist (**ii**). Reproduced from [38] with permission from the American Chemical Society. Copyright (2015). (**d**) Photograph (**i**) and IR image (**ii**) of WSPHS at the knee position of a human body after switching on the device at a DC voltage of 1.4 V. Reproduced from [62] with permission from the American Chemical Society. Copyright (2016). (**e**) Photograph (**i**) and IR image (**ii**) of strip–shaped TRHs woven into textile after applying a voltage. Reproduced from [60] with permission from the Royal Society of Chemistry. Copyright (2017). (**f**) An exploded view schematic illustration of a thin, flexible, wireless system for generating programmable patterns of thermal sensation across large areas of the skin (**i**), photograph of thermal interfaces on the inner sides of a stretchable compression shirt at the chest, back, abdomen, lower arm (**ii**). Reproduced from [19] with permission from the National Academy of Sciences. Copyright (2023). (**g**) An exploded view schematic illustration of a wireless thermo-haptic stimulator system, showing its key components, including batteries, haptic actuators, electronics, thermoelectric modules, and encapsulation layers (**i**), and photographs of the system mounted on the upper arm, lower arm, upper leg, and lower leg (**ii**). Reproduced from [20] with permission from the National Academy of Sciences. Copyright (2024).

The finger is one of the most sensitive areas to tactile stimuli on the human body [134], making it a focal point for initial research on delivering thermal stimuli. A ring was proposed, incorporating multiple TEDs designed to deliver combinatorial thermal patterns (Figure 8a). Despite its early–stage design and relatively rough form factor, participants were able to identify two distinct groups of thermal patterns with an average accuracy exceeding 80% [21]. The “ThermoVR” provides integrated thermal haptic feedback through a head–mounted display (HMD), enhancing immersive experience in VR/AR environments [130]. The prototype system incorporates five TEDs: three positioned on the forehead and two on the face. Additionally, three temperature sensors are strategically placed for accurate thermal monitoring. Evaluation results demonstrated a thermal perception accuracy of approximately 89.5% for cold stimuli and 68.6% for hot stimuli.

Glove–type WTDs offer a distinct advantage by significantly enhancing the user experience in immersive VR/AR environments through in–glove delivery of combined tactile and thermal stimuli to all fingers, which have a high density of cutaneous sensory receptors. The thermal display glove system incorporates multiple TEDs, piezoelectric sensors, an interface board, and computer software (Figure 8b). A wearable inertial measurement unit (IMU) enables virtual hand positioning, allowing users to interact with virtual objects while receiving thermal feedback via wireless communication between the interface board and the computer [26]. The “ThermAirGlove (TAGlove)” features another type of cotton glove equipped with five inflatable airbags on the fingers and palm, integrated with a closed–loop pneumatic thermal control system [27]. User perception experiments demonstrated that “TAGlove” could deliver five distinct levels of thermal sensation, ranging from very cool to very warm, and enable material identification among foam, glass, and copper with an average accuracy of 87.2%.

Resistive heaters made from metal nanowires offer enhanced versatility for improved wearability across various body locations. Patterning Ag NWs composites into serpentine–mesh structures allows for the conformal lamination of devices onto curvilinear joints, such as the wrist and knee. A stretchable mesh heater measuring 14 × 6.5 cm^2^, designed to fit the average wrist size of adult subjects, was integrated with a custom–made electronic band (Figure 8c(i)). Powered by the battery integrated into the electronic band, the stretchable mesh generates heat evenly across its entire area while maintaining conformal contact with the joint during flexion and extension (Figure 8c(ii)). Test subjects reported no rashes or signs of irritation after wearing it for 12 h [38]. The aforementioned WSPHS (Figure 3g–i), a heating fabric integrating SHFs composed of composite fibers with Cu NWs, is demonstrated as a knee pad applied to the knee of a human body (Figure 8d), effectively heating the targeted area and showcasing its practical application [62]. A stretchable strip–shaped TRH, composed of a CNT sheet and silicon elastomer (Figure 4a–c), can be woven into commercial fabrics with various patterns (Figure 8e), demonstrating localized heating functionality and body–conformable mechanical properties [60].

An earable form factor equipped with multiple TEDs can deliver thermal stimuli to multiple points on the auricular skin [28]. The “ThermEarhook” prototypes feature a 3D–printed earhook frame integrated with 3, 4, or 5 TEDs. A pilot study was conducted using an Arduino–based thermal control system, with the “ThermEarhook” connected to a Surface Pro laptop via a USB cable. This study primarily investigated perception accuracy, response time, and user preferences for single– and multi–point thermal patterns applied to one or both ears. User–perception tests revealed that participants adjusted the hot and cold stimuli to perceivable and comfortable levels at each of the five points on the earhook. Another type of wearable form factor is a neoprene drysuit, measuring 4 × 4 cm^2^ and integrating 35 thermoelectric skins [132]. This suit demonstrates dual–mode capabilities, enabling self–powering for the circuit and multiple sensors attached to the wearer, as well as thermoregulation in underwater environments.

These and other WTDs, whether in accessory form factors or designed for specific body parts, effectively deliver thermal stimuli to targeted areas; however, scaling them into flexible, large–area systems remains in the early stages of development. A recent effort introduces a thermally controlled epidermal VR system (“t–eVR”) weighing 23.5 g (excluding the battery), with a total thickness of 3.5 mm and a coverage area of approximately 5221 mm^2^, supporting various skin–interfaced form factors for both large and small body areas (Figure 8f). The system integrates 16 (4 × 4) independently controlled thermal modules, each measuring 207 mm^2^ and separated by an edge–to–edge distance of 5 mm, with the unit thermal module configuration shown in Figure 7a. Perception tests conducted for heating areas of 2.1, 8.3, 18.7, and 33.2 cm^2^–by operating 1, 4, 9, and 16 heaters, respectively, demonstrate average perception accuracies of 75.0, 75.7, 75.9, and 81.8% on the forearm. This layout is strategically designed to account for the anatomical distribution and sensitivity of thermoreceptors across different body regions [19].

Another platform leverages combined arrays of thermal and haptic actuators in a spatially integrated, vertically coupled configuration, enabling high–resolution (one thermo–haptic unit per 2.3 cm^2^), programmable patterns of enhanced vibrational displacement and high–speed thermal stimulation across large body areas (Figure 8g). The system features an array of 15 individually addressable thermo–haptic stimulators, arranged with a 19 mm center–to–center separation, each incorporating a miniaturized eccentric rotating mass (ERM, 7 mm diameter) vibro–mechanical actuator bonded atop a small–scale thermoelectric cell (6 × 6 × 2.7 mm^3^) [20].

## 5. Applications of WTDs in Virtual and Augmented Reality

VR/AR applications utilizing WTDs enhance user immersion through either a single thermal interface at the skin or a combined thermal–haptic interface. These applications primarily investigate the WTDs with various form factors, often employing TEDs due to their ability to provide both heating and cooling with rapid response times and customizable fabrications features [135,136]. At the system–level, integrated setups typically incorporate both hardware and software components, including analog–to–digital conversion, automated signal transmission, and user interfaces with wired connections to display units [24,137,138]. Research in this field focuses on analyzing perceptual responses to thermal feedback in VR/AR spaces and evaluating the extent to which such feedback enhances user immersion and differentiation in virtual or augmented experiences.

**Figure 9:**
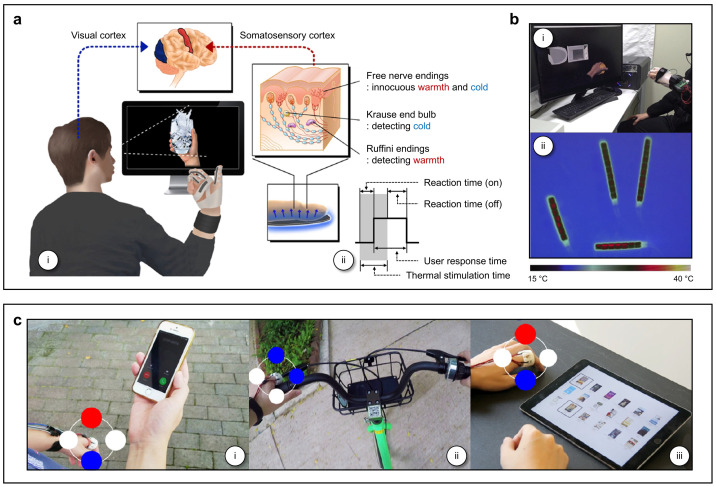
Applications of WTDs in virtual and augmented reality. (**a**) Illustration depicting the operation of the thermal display glove system interfaced with a virtual environment and the corresponding physiological processes (**i**), along with the definition of reaction time in response to thermal stimulation (**ii**). (**b**) Photograph of a thermal display glove system, showing a user grasping a heated object in a microwave (**i**) and the corresponding thermal imaging camera screen (**ii**). Reproduced from [26] with permission from the Springer Nature. Copyright (2020). (**c**) Photograph of a “Smart ring” demonstrating its application for notification (**i**), navigation (**ii**), and digital artifact comparison (**iii**). Reproduced from [21] with permission from the Elsevier. Copyright (2019).

The thermal display glove system [26] integrates with the VR environment and physiological processes, particularly the visual and somatosensory systems (Figure 9a(i)). A key aspect of the system is that the brain processes these two external stimuli as if they were from a real–world scenario, enhancing the sense of realism in the VR environment. Defining reaction times is a critical consideration, as delays were observed between the thermal stimulation and the user’s response during testing. The reaction times for stimulus onset and offset were defined as the latency between the activation/deactivation of the thermal stimulus and the corresponding user response, respectively (Figure 9a(ii)). When a user wearing the glove interacts with a hot object (e.g., a chicken in a microwave) within the VR space, they receive both visual and thermal stimuli from the display (Figure 9b).

The ring prototype, which incorporates multiple TEDs, utilizes the position of each TED for spatial mapping in navigation applications [21]. Thermal feedback, delivered through single–spot or combinatorial thermal patterns, has the potential to serve as a notification mechanism for incoming calls or messages, facilitate navigation functions, and assist in the comparative analysis of digital artifacts (Figure 8a and Figure 9c). Additionally, the device can function as an emotional interface by encoding positive emotions through the top–positioned TED and negative emotions through the bottom–positioned TED. Furthermore, hot stimulation can be employed as an alert mechanism for emergencies, unpleasant events, or to signal undesirable phone calls or messages [139,140].

Integrating wristband–type WTDs with a smartwatch enables several potential applications [25]. The “ThermalBracelet” prototype, for instance, explores haptic guidance through spatiotemporal thermal stimuli, where clockwise or counterclockwise patterns could indicate movement directions, such as going upstairs or downstairs about a target location. In a notification scenario, distinct thermal patterns could differentiate between various applications, such as multiple notifications from a calendar app versus a single notification from a messaging app. Furthermore, the device could convey weather conditions, delivering cold thermal feedback for rainy or cold days and warm thermal feedback for sunny or hot days. The HMD integrated with five TEDs enables users to experience spatial and immersive thermal sensations on their face [130]. In one game scenario, players can perceive cold through immersive thermal feedback simulating a winter night. The application further enhances immersion by providing directional cues that allow players to explore different thermal environments, such as warm weather or the intense heat of a campfire, depending on the virtual location.

**Figure 10:**
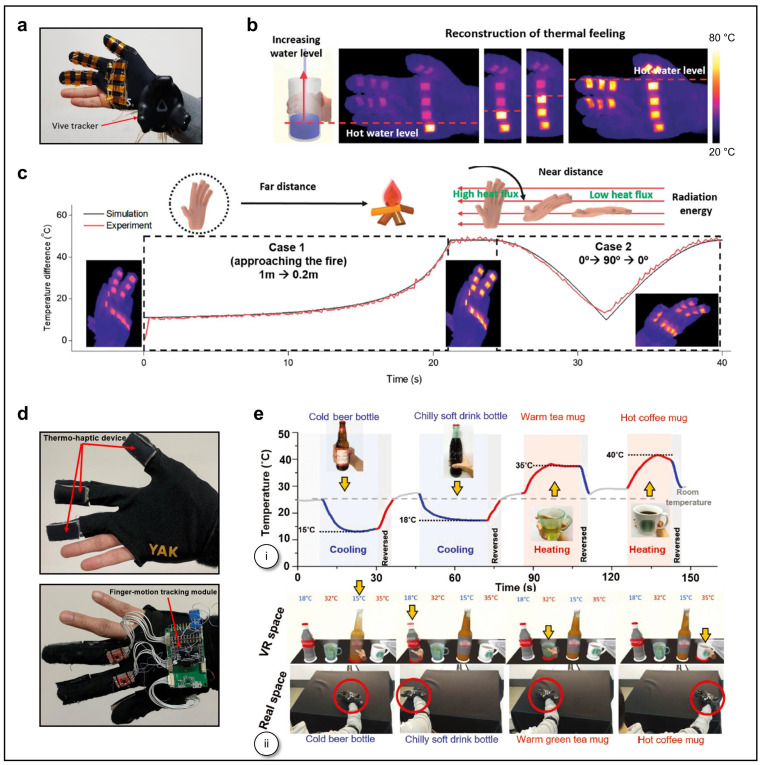
Reconstruction of artificial thermal sensation in VR space. (**a**) Photograph of a nylon glove incorporating 12 heaters, a motion tracker, inertial measurement units (IMUs), and strain sensors. (**b**) Virtual image and IR images illustrating temperature changes in both simulation and experimental results when touching a cup filled with water at 50 °C. (**c**) Temperature variations in simulation and experimental results when approaching a heat source (Case 1) and changes in hand angle (Case 2). Reproduced from [31] with permission from the Royal Society of Chemistry. Copyright (2020). (**d**) Photographs of the palm and backside of the VR glove that integrates thermo–haptic devices and a finger–motion tracking module. (**e**) The temperature profile shows changes in temperature based on the virtual scenario of touching various objects (**i**), and the two sets of snapshots correspond to the simultaneous motion of the hand in VR and real space, respectively, when touching objects at various temperatures, such as a cold beer bottle, a chilly soda bottle, a mug of warm green tea, and a mug of hot coffee (from left to right) (**ii**). Reproduced from [29] with permission from Wiley–VCH. Copyright (2020).

Recent glove–type WTDs that utilize both resistive heating mechanisms and the Peltier effect have successfully demonstrated the reconstruction of artificial thermal sensations in VR space [29,31]. A nylon glove with 12 heaters made from Cu NWs–PUA electrodes, a motion tracker, IMUs, and strain sensors is used to replicate various virtual scenarios (Figure 3d–f and Figure 10a). Virtual and IR images in Figure 10b show that each heater is independently controlled to achieve different desired temperatures over time, delivering varying heat flux depending on the temperature of hot (50 °C) water. During a 30 s control, a mean absolute percentage error (MAPE) of less than 3% between the desired and actual heater temperatures confirms the high reproducibility of the heaters. Another experiment evaluates the replication of heat transfer when a heat source approaches (Case 1) and when the heat source or hand angle changes (Case 2) (Figure 10c). The results show that the temperature variations with respect to the distance and angle between the heating source and the palm are accurately described, with a MAPE of less than 4%, validating the accuracy of the virtually reproduced thermal radiation [31].

Another nylon VR glove prototype that integrates flexible TEDs enables the reproduction of virtual thermal sensations when touching objects at various temperatures (Figure 6g–i and Figure 10d). In situ data collection during a virtually realized scenario, where the user grasps objects such as a cold beer bottle, a chilly soda bottle, a mug of warm green tea, and a mug of hot coffee, shows that the proportional–integral–derivative (PID) control system closely reproduces the temperature changes in the objects with minimal fluctuation (Figure 10e). Conversely, when the fingers are detached from the objects, the temperature rapidly returns to room temperature after touching both cold and warm objects [29].

Specifically, skin–integrated technologies serves as a basis for studying the characteristics of thermoreceptors, including stimulation area, magnitude, duration, and the rate of temperature change. In this context, the following section focuses on studies that investigate the potential for delivering programmable, spatiotemporal thermal patterns over a large area of the human body, perception studies, and their applications for VR/AR.

**Figure 11:**
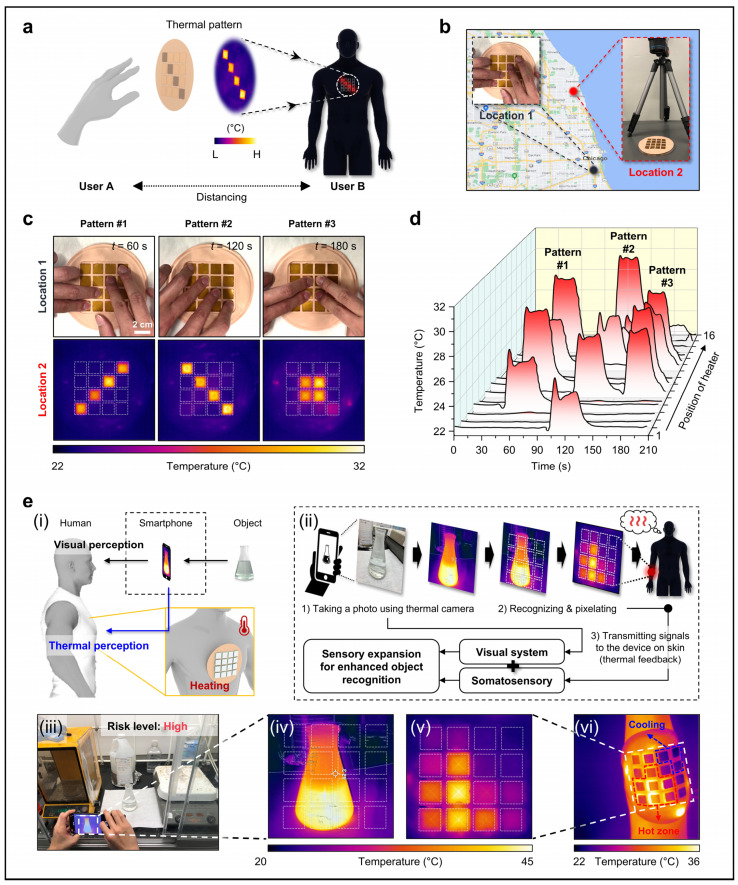
Remote communication and sensory expansion by thermal mirroring. (**a**) Conceptual illustration of device–to–device communication between users at separate locations. (**b**) Photographs of measurement setup at two locations (~20 km apart) where a pattern of finger touch defines a corresponding spatiotemporal pattern of temperature on a device at location 1, and the temperature data transmitted from location 1 is reproduced in real–time at a device at location 2. (**c**) Photographs of patterns of touch #1–3 applied to the device at location 1 and IR images of corresponding thermal patterns #1–3 generated by the device at location 2. (**d**) Temperature profile of spatiotemporal thermal patterns measured at location 2. (**e**) Conceptual illustration of sensory expansion (**i**), flow chart for remote object recognition through automated pixelation and transmission of temperature data (**ii**), photograph of a flask in which an exothermic chemical reaction occurs (**iii**), corresponding IR images of the flask (**iv**), device with related thermal feedback (**v**), and thermal interface of the device on the forearm (**vi**). Reproduced from [19] with permission from the National Academy of Sciences. Copyright (2023).

The aforementioned “t–eVR” systems (Figure 7a and Figure 8f) enhance remote social interactions by enabling the long-range delivery of spatiotemporal thermal patterns for heating and cooling, controlled through multichannel wireless communication between users [19]. In the experimental setup, one device captures and transmits temperature distributions generated by fingertip touch at location 1, while another device reproduces this thermal information at location 2 (~20 km apart) (Figure 11a,b). The sequence of IR images of the device (Figure 11c) and the corresponding temperature profiles for each thermal module at location 2 (Figure 11d) validate the system’s operation. Another VR application involves real–time sensory expansion for remote thermal recognition using inputs from an IR camera (Figure 11e(i)). In this demonstration, a portable IR imaging system detects temperature distributions of a remote object, providing a mechanism to prevent burns. The captured thermal data are wirelessly transmitted to the “t–eVR”, which then adjusts its operation accordingly, as illustrated in the flow chart (Figure 11e(ii)). An IR image captured at a resolution of 160 × 120 pixels using a mobile IR camera is re–pixelated in real–time to a 4 × 4 grid. The average temperature of each pixel serves as the set point for a corresponding thermal module mounted at a desired body location. A representative example of this application is remote temperature sensing for laboratory safety (Figure 11e(iii–vi)).

**Figure 12:**
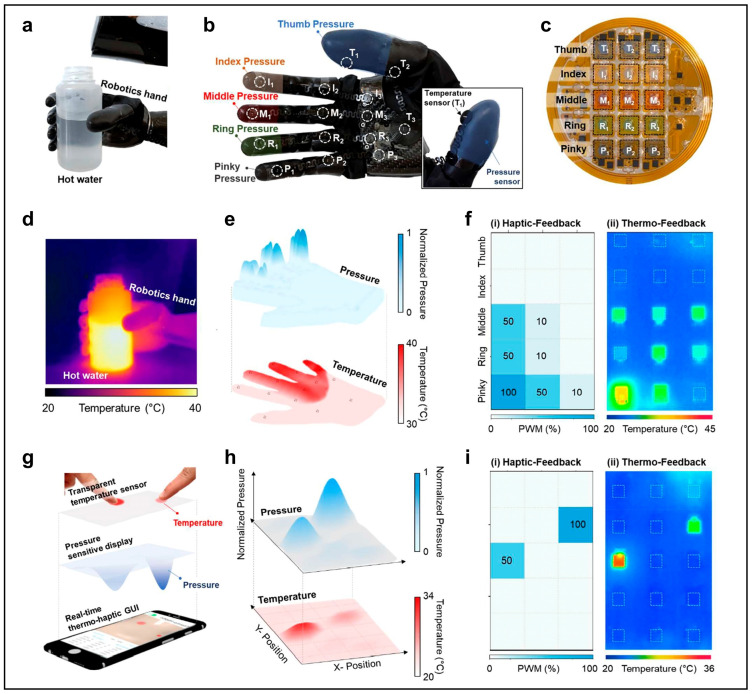
Reconstruction of tactile sensation through a thermo–haptic system. (**a**–**c**) Photographs of a robotic hand gripping a bottle of hot water (**a**); placements of pressure and temperature sensors on the robotic hand (**b**); and a schematic map of thermo–haptic stimulators, with each stimulator corresponding to a specific position on the fingers of the robotic hand. (**d**) IR image of a robotic hand grasping a hot bottle. (**e**) Measured distributions of pressure and temperature in the robotic hand while gripping a hot bottle. (**f**) Tactile reconstruction results of the robotic hand; (**i**) distribution of the pulse width modulation (PWM) duty cycle, and (**ii**) IR image of the thermo–haptic system providing haptic and thermal feedback. (**g**) Exploded view schematic illustration of a tactile display integrated with a transparent temperature sensor array. (**h**) Measured distribution of pressure and temperature across a multi–touch tactile display. (**i**) Tactile reconstruction results of the tactile display; (**i**) distribution of PWM duty cycle, and (**ii**) IR image of the thermo–haptic system providing haptic and thermal feedback. Reproduced from [20] with permission from the National Academy of Sciences. Copyright (2024).

Combined arrays of thermal and haptic actuators are essential for tactile reconstruction, enabling simultaneous temperature and pressure feedback [20]. This study explores thermo–haptic integration in two systems: a robotic prosthetic hand and a pressure–sensitive mobile display. Both systems incorporate pressure and temperature sensors to generate control signals for thermo–haptic stimulation, facilitated by real–time wireless communication. In the robotic hand, 15 thermistors co–integrated with pressure sensors detect thermal and pressure distributions of grasped objects (Figure 12a,b). Sensor data directly map to thermo–haptic actuators (Figure 12c), with temperature signals controlling TEDs, while pressure signals regulate ERM actuators via pulse-width modulation (PWM). When gripping a hot water–filled bottle (Figure 12a,d), pressure values (0–110 kPa) are converted into vibration intensities, with PWM duty cycles of 10%, 50%, and 100% corresponding to 20, 60, and 80 kPa, respectively (Figure 12f(i)). Thermal feedback is directly reproduced (Figure 12f(ii)). A 5 × 3 transparent temperature sensor array enables a pressure–sensitive touchscreen (Figure 12g). Pressure (1–400 steps) maps to vibration intensities via PWM, with 10%, 50%, and 100% corresponding to 10, 20, and 30 kPa, respectively (Figure 12i(i)). Thermal differences are captured in the IR image (Figure 12i(ii)).

## 6. Conclusions and Future Outlook

In this review, we highlighted recent advancements in thermal technologies for virtual and augmented reality (VR/AR) applications. Various resistive heating elements capable of generating thermal energy were examined. While resistive heaters are widely used for their simplicity and efficiency, they are limited to single–function heating without cooling capabilities. Advances in resistive heating materials, such as metal nanowires, improved oxidation resistance through nano–coatings, and enhanced mechanical stability, could unlock their full potential for industrial applications of wearable thermal devices (WTDs). Challenges that liquid metals must address include high cost, oxidation, encapsulation issues, and complex fabrication processes, which hinder their large–scale, low–cost adoption. Thermoelectric devices (TEDs) offer precise temperature control, fast thermal response, and power generation capabilities, yet their thick and rigid structures pose challenges for integration into soft, skin–conformal WTDs. Additionally, high costs and integration difficulties remain barriers to widespread use. Passive cooling elements emerge as an energy–efficient alternative, utilizing evaporation or radiative cooling, while their active controllability remains a challenge. Innovative engineering approaches will be essential to overcoming these limitations and for continued development of this field. In addition to the heating and cooling mechanisms described above, the selection of multifunctional materials for integration into WTDs is a critical consideration. For instance, shape–memory polymers can be synergistically combined with thermal actuators or sensors to enable programmable, high–density informatic patterns of thermal or haptic stimuli [141,142].

We also explored various wearable form factors of WTDs and their applications in VR/AR environments. While most existing WTDs primarily target the hands, enabling rapid thermal feedback at any location of the body without restriction could serve as the basis for more immersive VR/AR experiences. This extends beyond current systems that integrate only a limited number of actuators into garments. Achieving large area coverage with conformal, comfortable, and easily wearable designs—while considering thermoreceptor distribution and thermal perception—will be critical for advancing next-generation VR/AR technologies.

Beyond the aspects discussed in this review, future research should also focus on thermal sensing technologies and their seamless integration with WTDs. Wearable thermal sensors are critical components for VR/AR applications, enabling real–time monitoring and closed–loop control of thermal stimuli. Significant advancements have been made in thermal sensor technologies, which operate based on thermoresistive, pyroelectric, and thermoelectric mechanisms [143,144]. For high–performance WTDs, thermal sensors should exhibit fast response time, continuous monitoring capabilities for localized temperature changes, high accuracy and sensitivity, a broad sensing range, and long–term stability. Neuromorphic sensing mechanisms, inspired by human multisensory processing, offer a promising integration strategy for advanced WTDs [145,146,147]. Furthermore, these sensors must function without causing discomfort to the user, ensuring seamless integration into wearable systems for an enhanced immersive experience in VR/AR environments [39]. Ultimately, research into thin, soft, and skin–conformal WTDs, in conjunction with advancements in VR/AR technologies, will redefine human interaction with the digital world, driving innovation across multiple industries.

## Figures and Tables

**Table 1:** Summary of some wearable thermal devices (WTDs) utilizing resistive heating.

Materials and Structure	Temperature Range	Electrical Input	Lateral Size and Form Factor	Ref.
CNT ^1^ and Ag NWs on PET ^2^ substrate	25–110 °C	5–15 V	4 × 4 cm^2^	[46]
Ligand exchanged Ag NWs in SBS ^3^ elastomer	25–60 °C	0.5–1.0 V	4 × 4 cm^2^, stretchable (60%), wrist–wearable	[38]
CNT on Ecoflex	25–268 °C	10–50 V	4.5 × 3.0 cm^2^, stretchable (400%)	[47]
Carbonized weft–knitted fabric	20–140 °C	~3.5 V	4.5 × 4.5 cm^2^, stretchable (70%), knee–wearable	[48]
Kirigami–patterned Al paper on Ecoflex	22–43 °C	0.3–1.5 V	14.5 × 12 cm^2^, stretchable (400%), wrist–wearable	[49]
PEDOT ^4^ coated on the fabrics	21–44 °C	3–6 V	1 × 1 cm^2^, integrated with a glove	[50]
Ag NW–cloth, CNT–cloth	21–55 °C	0.6–1.2 V (Ag NW–cloth) 8–16 V (CNT–cloth)	1 × 1 in. ^2^, integrated with a glove	[51]
Ag NW in PDMS ^5^ film	20–150 °C	2–10 V	Stretchable (60%), wrist–wearable	[52]
Liquid metal in PDMS film	25–95 °C	0.5–3.5 V	~3 × 3 cm^2^, stretchable (>100%), kneepad	[53]
Ag NW with PEDOT:PSS ^6^	20–110 °C	3–6 V	5 × 6 cm^2^	[54]
Ag NW with rGO ^7^	68–230 °C	5–15 V	N/A *	[55]
Cu–Ni NW in PET film	20–106 °C	3–15 V	4 × 2 cm^2^	[56]
Ag NW–Clay on PET film	40–110 °C	3–7 V	25 × 20 cm^2^	[57]
Cu NW in PUA ^8^ film	20–120 °C	1–7 V	1 × 1 cm^2^, stretchable (>50%), integrated with a glove	[31]
GF ^9^–embroidered cotton fabric	50–424 °C	0.9–4.7 V	Stretchable (70%), wrist–wearable	[58]
Ni–EGaIn ^10^ electronic tattoo	25–66 °C	0.1–0.5 A	4.5 × 7 cm^2^, integrated with a glove	[59]
CNT on thermochromic silicon elastomer	45–95 °C	4–8 V	2 × 0.5 cm^2^, stretchable (100%)	[60]
CB/PU ^11^ printed textiles	30–85 °C	2.5–20 V	4 × 3 cm^2^	[61]
Cu NW/PE ^12^ yarn on fabric	25–150 °C	0.9–6 V	~8 × 6 cm^2^, stretchable (40%), integrated with a cloth	[62]
CNT HHF ^13^ on textile	38–60 °C	5–9 V	15 × 7 cm^2^, stretchable (140%), integrated with a textile	[63]
EGaIn sandwiched in silicone	25–89 °C	1–6 V	6 × 7 cm^2^, stretchable (100%)	[64]

^1^ CNT: carbon nanotube. ^2^ PET: polyethylene terephthalate. ^3^ SBS: poly(styrene–*b*–butadiene styrene). ^4^ PEDOT: poly(3,4–ethylenedioxythiophene). ^5^ PDMS: polydimethylsiloxane. ^6^ PSS: polystyrene sulfonate. ^7^ rGO: reduced graphene oxide. ^8^ PUA: polyurethane acrylate. ^9^ GF: graphene fiber. ^10^ EGaIn: eutectic gallium indium. ^11^ PU: polyurethane. ^12^ PE: polyethylene. ^13^ HHF: hierarchically helical fiber. * N/A: not available.

**Table 2:** Summary of some wearable thermal devices (WTDs) utilizing Peltier effect.

Materials and Structure	Temperature Range	Electrical Input	Lateral Size and Form Factor	Ref.
Bi_2_Te_3_ based TE device with Ecoflex/Ag flake composite	~15–35 °C	~0.2–1.5 A	2.7 × 2.7 cm^2^, 4 × 4 arrays, stretchable (230%), integrated with a glove	[29]
Bi_2_Te_3_ based TE device	~22–35 °C	~1–2.4 A	1.6 × 1.6 cm^2^, 6 × 6 arrays	[115]
Bi_2_Te_3_ based TE device with AIN Ecoflex/AIN powder composite	~25–42 °C (from skin temperature)	~−60–140 mA	5 × 5 cm^2^, 12 × 12 arrays, flexible (BR ^1^ ~40 mm), integrated with an armband	[30]
BST based TE devices (Bi_0.5_Sb_1.5_Te_3_ for *p*–type and Bi_2_Te_2.7_Se_0.3_ for *n*–type) with EGaIn/PDMS	~5.4 K (human body cooling)	N/A *	4.25 × 3.5 cm^2^, 6 × 5 arrays, flexible (BR ~29.35 mm)	[116]
Bi_2_Te_3_ based TE device with Ag flake/EGaIn/Ecoflex composite, thermochromic liquid crystal coating	~3–40 °C	~1.5–0.6 A	9 × 5 arrays, flexible (BR ~2 cm)	[117]
Bi_2_Te_3_ based TE device	~30–33 °C	N/A	Ring cases (6–11 US sizes), 4 or 6 or 8 TE devices	[21]
Bi_2_Te_3_ based TE device	N/A	N/A	Wrist band (67 × 42 × 27 mm^3^), 2 TE devices	[22]
Bi_2_Te_3_ based TE device with airbags	28–40 °C	N/A	2 TE devices (40 × 40 mm^2^) in each hot and cold chamber, integrated with a pneumatic glove	[27]
Bi_2_Te_3_ based TE device	N/A	N/A	Earhook cases, 3 or 4 or 5 TE devices	[28]
Bi_2_Te_3_ based TE device with Ecoflex	15–40 °C	0.67 A	75 × 5 mm^2^ (middle finger), flexible (BR ~20 mm), integrated with a display glove	[26]

^1^ BR: bending radius. * N/A: not available.
